# Plasma-activated medium as adjuvant therapy for lung cancer with malignant pleural effusion

**DOI:** 10.1038/s41598-020-75214-2

**Published:** 2020-10-23

**Authors:** Yi-Jing Cheng, Ching-Kai Lin, Chao-Yu Chen, Po-Chien Chien, Ho-Hsien Chuan, Chao-Chi Ho, Yun-Chien Cheng

**Affiliations:** 1 Department of Mechanical Engineering, College of Engineering, National Chiao Tung University, EE465, 1001 University Road, 30010 Hsin-Chu, Taiwan; 2 Department of Internal Medicine, National Taiwan University Hospital, Taipei, Taiwan; 3 Department of Internal Medicine, National Taiwan University Hospital Hsin-Chu Branch, Hsin-Chu, Taiwan; 4 Department of Medicine, National Taiwan University Cancer Center, Taipei, Taiwan; 5 Department of Surgery, National Taiwan University Hospital Chu-Tung Branch, Hsin-Chu, Taiwan

**Keywords:** Cancer therapy, Biomedical engineering

## Abstract

This study compared effects of plasma-activated medium (PAM) with effects of conventional clinical thermal therapy on both lung cancer cells and benign cells for management of malignant pleural effusion (MPE). For MPE treatment, chemotherapy, photodynamic therapy, and thermal therapy are used but caused systemic side effects, patient photosensitivity, and edema, respectively. Recent studies show that plasma induces apoptosis in cancer cells with minor effects on normal cells and is cost-effective. However, the effects of plasma on MPE have not been investigated previously. This study applied a nonthermal atmospheric-pressure plasma jet to treat RPMI medium to produce PAM, carefully controlled the long-life reactive oxygen and nitrogen species concentration in PAM, and treated the cells. The influence of PAM treatment on the microenvironment of cells was also checked. The results indicated that PAM selectively inhibited CL1–5 and A549 cells, exerting minor effects on benign mesothelial and fibroblast cells. In contrast to selective lethal effects of PAM, thermal therapy inhibited both CL1–5 and benign mesothelial cells. This study also found that fibroblast growth factor 1 is not the factor explaining why PAM can selectively inhibit CL1–5 cells. These results indicate that PAM is potentially a less-harmful and cost-effective adjuvant therapy for MPE.

## Introduction

Lung cancer is one of the leading causes of cancer-related deaths worldwide^1^, whose 5-year survival rate remained lower than 25% in 2016^2^. The outcome of the disease depends on staging, and so proper staging must be performed to determine the optimal treatment plan^3^. A previous guideline classified lung cancer with pleural seeding as late-stage disease due to the inoperable condition. In the normal condition, only a minimal amount of pleural effusion within the pleural space allows for movement of the lung relative to the chest wall during breathing^4^. Malignant cells in the pleural space may change the vascular permeability and impede resorption, causes the leakage of excessive fluid into the pleural space. The presence of a space-occupying liquid in the pleural space results in the compression of normal lung and limits the movement of respiratory muscles, such as diaphragm^5^. For these reasons, dyspnea is the most common symptom in patients with massive malignant pleural effusion (MPE). Porcel et al. reported that 40% of patients with lung cancer develop pleural effusions during their disease course, and that the survival rate was much lower for patients with MPE^6^.

The conventional treatment for patients with MPE is palliative, consisting of repeating thoracentesis for symptom relief, talc pleurodesis for decreasing the production of the effusion, or combining chemotherapy for systemic control. Radiotherapy is rarely administered to these patients due to the pulmonary toxicity of its hemithoracic application^7^. New advanced techniques have been developed for lung cancer patients with isolated pleural metastasis. Previous in vitro and in vivo studies have found that heating may enhance the cytotoxic effects of cisplatin^8,9^. Combining cisplatin and hyperthermia with pleural perfusion resulted in excellent local control and better survival for lung cancer with pleural seeding^10,11^. Lung edema may occur in some patients after applying hot-fluid lavage within the pleura space due to the induction of an inflammatory reaction. Cisplatin may diffuse into the circulatory system via the pleura to cause systemic side effects such as nausea, vomiting, and nephrotoxicity.

Photodynamic therapy (PDT) uses laser light to activate the photosensitizer and contribute to cytotoxic activity to tumor cells. Combining PDT and video-assisted thoracoscopic surgery provides good local control of malignant pleural tumors^12,13^. However, PDT has several disadvantages, including skin and eye sensitivity to light, pain to nearby healthy tissue, and high cost.

Plasma is an ionized gas composed of positive/negative ions, electrons, radicals, uncharged (neutral) atoms and molecules, and UV photons^14^. Plasma has been shown to generate both short- and long-lived molecules such as reactive oxygen and nitrogen species (RONS) mainly from the oxygen and nitrogen present in atmospheric air and solutions^15,16^. Nonthermal atmospheric-pressure plasma jet (NTAPPJ) treatments have been shown to be effective in inducing apoptosis in a broad range of cancer cell types with few effects on normal cells^17–21^. Keidar et al. reported that nonthermal plasma treatment selectively inhibits cancer cells in vitro without damaging normal cells^18^. A prepared medium irradiated by NTAPPJ, termed plasma-activated medium (PAM) and other plasma-activated solutions exerts similar effects^22,23^. RONS, such as H_2_O_2_, can induce apoptosis in some cancer cell lines^24–26^. Other RONS that can possibly affect cancer cells include O^27,28^, ^·^OH^27–29^, NO^30–33^, ONOO^–^^33–35^, N_2_^27^, N^+^^27^, Ar^27^, Ar^+^^27^, Ar_2_^+^^27^, H_2_O_2_^29^, and O_2_^–^^29^. Additionally, NO can both improve and inhibit the apoptosis of cells via mechanisms that are not yet clear^30^. Besides, there are recent reports that the co-treatments of NTAPPJ and other anti-tumor medicines or nanoparticles contribute to synergistically therapeutic effects^36,37^. Plasma can also be self-adaptive to adjust plasma interaction with cells^38^.

Hence, NTAPPJ and PAM may be a novel and convenient adjuvant therapy for cancer^38–40^. RONS which selectively induce apoptosis more in cancer cells than in normal cells have comparatively short lifetimes and thereby possibly minimizing the systemic side effects. Also, a plasma system is cost-effective (costing about USD 1,000 per system). However, the possibility of applying plasma in the therapy for lung cancer with MPE remains obscure.

In this study, we measured the RONS concentration in the PAM and compared the effects of the PAM at different dosages on both adenocarcinoma cells and benign cells. For future clinical applications, we compared the PAM treatment and hyperthermia therapy on cells. We also discussed the microenvironment change of cancer after plasma treatment. The purpose of this study was to determine the antitumor effects from PAM in order to optimize the conditions of plasma therapy for MPE.

## Methods

### Setup and characterization of NTAPPJ

#### The NTAPPJ

**Figure 1:**
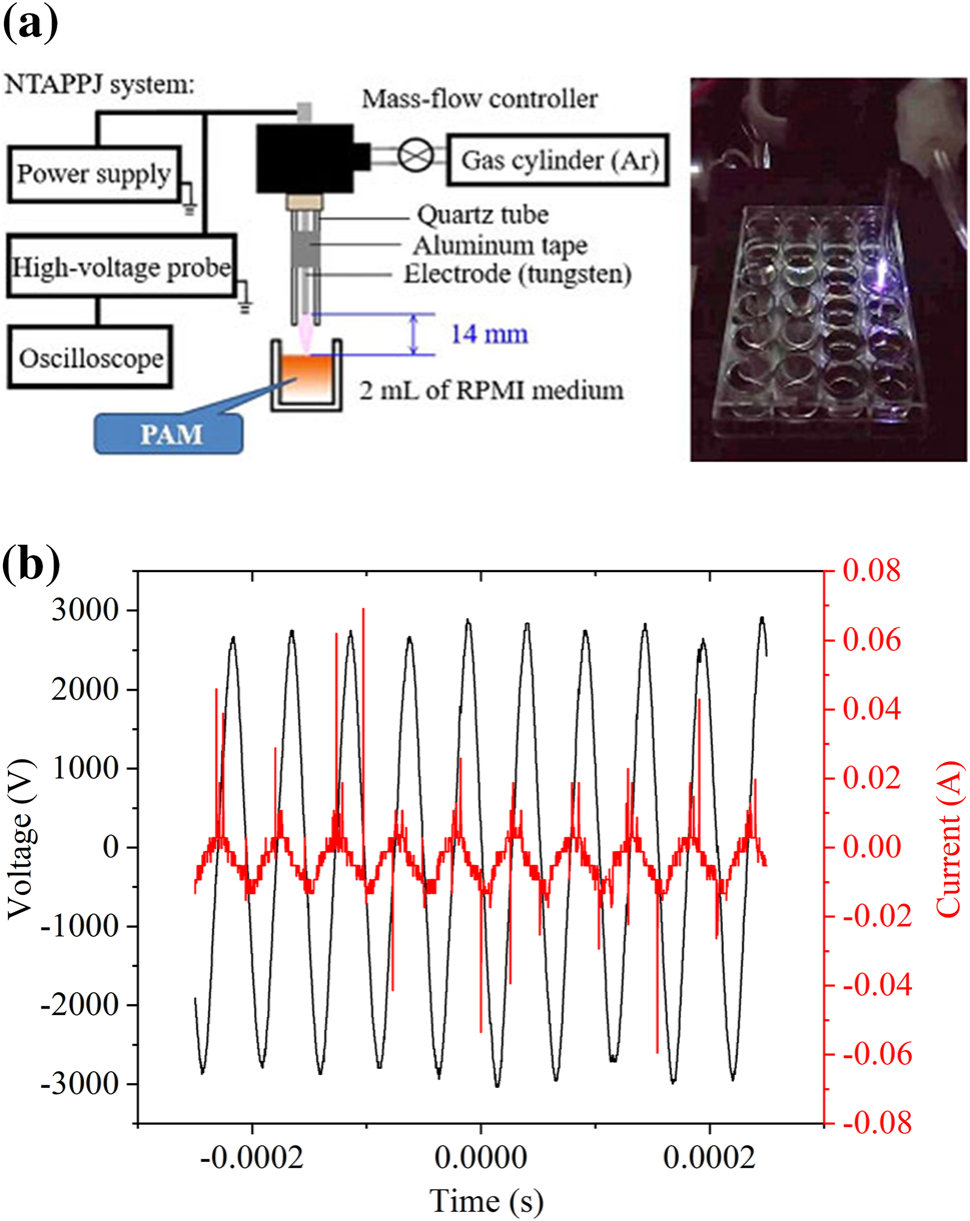
(**a**) Schematic and operation of PAM preparation. (**b**) Voltage and current waveforms of the plasma system.

The experimental NTAPPJ setup in this study and the preparation of PAM are shown in Fig. 1a. The powered electrode was made of tungsten. The grounded electrodes comprised aluminum tape installed outside a quartz tube surface. The working gas was Ar (99.99%), which flowed at a rate of 3 L/min into the quartz tube via a mass flow controller. The plasma was ignited by a portable AC power source whose sinusoidal output as monitored by an oscilloscope was 4 kV at a frequency of 19.5 kHz Fig. 1b. The holder of the jet was fabricated using ABS material by a 3-D printer (D-Force V3, Synmao Technology, Taipei, Taiwan). PAM was obtained by exposing 2 mL of Roswell Park Memorial Institute 1640 (RPMI) medium (Gibco, Sigma, Missouri, USA) in a 24-well plate to NTAPPJ. The powered electrode and liquid surface were separated by 14 mm.

The species present in the postdischarge jet region was identified using the optical emission spectrum (OES). The intensities of the NTAPPJs were measured at 10 mm from the outlet of the jet using a monochromator (Acton SP2500, Princeton Instruments, New Jersey, USA) with a photomultiplier tube (R928, Hamamatsu Corporation, New Jersey, USA) in air or 2 mm above the interface between the plasma and RPMI medium.

#### Measurement of ^·^OH radicals in the NTAPPJ-treated solution

First the terephthalic acid (TA) solution was prepared, comprising 2 mM TA (Sigma) and 5 mM NaOH (Merck, Darmstadt, Germany), and 2 mL of this solution was put into a 24-well plate and then exposed to the NTAPPJ for 0, 10, 20, 30, 40, 50, 60, and 120 s. In the solution, the generated ^·^OH radicals reacted with TA to become hydroxyterephthalic acid (HTA). Then 4 mL of the NTAPPJ-treated solution with generated HTA was loaded into a quartz cuvette. The HTA was excited at 310 nm, and the fluorescence emitted at 425 nm was measured by an optical emission spectrometer (USB2000+, Ocean Optics, Edinburgh, UK) and converted to the ^·^OH-radical concentration using a standard curve.

#### Preparation of PAM and measurement of its H_2_O_2_ content

PAM is prepared by treating RPMI medium with NTAPPJ. However, the RONS generated by plasma can be influenced by the humidity of the working gas, the constituents of the ambient air, and even turbulence in the plasma jet. To precisely control the RONS concentration of PAM applied to the cells, we controlled the temperature and humidity in the laboratory as well as the humidity of the working gas. In addition, before each experiment the concentration of the H_2_O_2_ generated by the NTAPPJ in PAM was measured to check for the concentration of reactive oxygen species (ROS), since H_2_O_2_ is an ROS that is easy to measure. The 100% PAM was prepared by treating 2 mL of RPMI medium without phenol red in a 24-well plate with NTAPPJ for 120 s to ensure that the increase in the H_2_O_2_ concentration entered a plateau. Afterwards, the following PAM-RPMI mixtures were produced by serially diluting the PAM using untreated RPMI medium: 0%, 10%, 20%, 30%, 40%, 50%, 60%, 70%, 80%, 90%, and 100%. Finally, the cells were treated with the PAM-RPMI mixtures but not the RPMI medium treated with the NTAPPJ for different times. These processing steps meant that the RONS concentration used to treat the cells could be precisely controlled.

The H_2_O_2_ concentration in PAM was measured using an Amplex Red analysis kit (Thermo Fisher Scientific, Massachusetts, USA). Two milliliters of RPMI medium without phenol red was loaded into a 24-well plate and exposed to the NTAPPJ for 0, 10, 20, 30, 40, 50, 60, and 120 s to produce PAM. Aliquots (50 μL) of PAM were taken to react with Amplex Red in the dark for 30 min. The absorbance of the generated resorufin was measured at 570 nm using a microplate reader (Anthos 2020, Biochrom, Cambridge, UK), and the H_2_O_2_ concentration was calculated using a standard curve. The H_2_O_2_ concentration of the PAM-RPMI mixtures was measured using the same method.

#### Measurement of nitrite ions in PAM

The concentration of the nitrite ions in PAM was measured to check the generation of reactive nitrogen species (RNS) with NTAPPJ treatment. Nitrite ions were measured using a water analyzer (DPM-MT-E, Kyoritsu, Tokyo, Japan) with a nitrite assay kit (WAK-NO_2_(C), Kyoritsu) that implemented the Griess test. The PAM-RPMI mixtures were prepared as described in Sect. 2.1.3. Four-milliliters of PAM-RPMI mixtures was added to the nitrite assay agent and allowed to stand for 3 min. The color intensity was measured at 525 nm, and then the concentration of nitrite ions was calculated using a standard curve.

### Effects of PAM on cells

#### Cell culture

Benign mesothelial cells from MPE were consecutively collected from lung cancer patients at National Taiwan University Hospital (NTUH). Normal fibroblasts and cancer-associated fibroblasts (CAFs)^41^ from lung cancer surgical specimens were collected from early stage lung cancer patients at NTUH. The CAFs were identified with morphology and α-smooth muscle actin (α-SMA) staining^42^. The study of these three cell types was approved by the Institutional Review Board of the NTUH Research Ethics Committee (approval number: 200804019R and 200912012R). All the patients signed informed consent before collection of specimens. All methods in this study were performed in accordance with the relevant guidelines and regulations. The CL1–5 and A549 cell lines were also obtained from NTUH.

Two lung adenocarcinoma cell lines (CL1–5 and A549) and the three noncancer cells (benign mesothelial cells, normal fibroblasts and CAFs) were cultured in RPMI medium supplemented with 10% FBS (Gibco, Sigma), 100 U/mL penicillin, and 100 mg/mL streptomycin (Corning, New York, USA) at 37 °C in a 5% CO_2_ humidified incubator.

#### Cell growth assay

In the cell growth assay, cells were seeded on 96-well plates at 2 × 10^4^ cells/well. After 1 day, the cells were treated with PAM-RPMI mixtures containing PAM at proportions ranging from 0 to 100% and maintained at 37 °C for 2 h. The PAM-RPMI mixtures were replaced by 100 µL of fresh medium and the cells were further cultured at 37 °C. Cell numbers were quantified on days 0, 2, 4, 6, and 8 by counting trypsin-detached cells after applying trypan blue staining. The relative growth was determined by cell counts, and dead cells were also enumerated.

#### Cell viability assay

In the cell viability assay, cells were seeded on 96-well plates at 2 × 10^4^ cells/well. After allowing 1 day for cell attachment, cells were treated with PAM-RPMI mixtures containing PAM at proportions ranging from 0 to 100%. The plates were then left to stand at 37 °C and 43 °C separately for 2 h. The PAM-RPMI mixtures were then replaced by 100 µL of fresh RPMI medium. Cells were further cultured at 37 °C for 1 more day. The viability of cells was measured using a 3-(4,5-cimethylthiazol-2-yl)-2,5-diphenyl tetrazolium bromide (MTT) assay (M6494, Thermo Fisher Scientific). The treated cells were cultured in MTT solution at a concentration of 0.5 mg/mL for 6 h.

#### Scratch assay

A scratch was made in the confluent cells cultured in 24-well plates at a time defined as time 0 (*t*_0_). The cells were then treated for 2 h with PAM-RPMI mixtures containing PAM at proportions ranging from 0 to 100%. After replacing the PAM-RPMI mixtures with fresh medium for 24 h, the cells were washed with PBS, fixed with 4% formaldehyde for 20 min, permeabilized with 70% methanol for 10 min, and stained with 0.5% crystal violet for 20 min. After washing the cells again, images were obtained using a digital microscope camera (DFC420 C, Leica, Wetzlar, Germany). The coverage area relative to the cell-free area at *t*_*0*_ was quantified using ImageJ software.

#### Annexin V staining assay

Cells were treated with and without PAM at 37 °C for 2 h and further cultured for 18 h. The apoptosis caused by PAM treatment was detected through phosphatidylserine binding with Alexa Fluor 488 Annexin V/Dead Cell Apoptosis kit (Thermo Fisher Scientific, USA) by following the standard procedures. Briefly, the treated cells were harvested, washed in PBS and resuspended in 100 µL annexin-binding buffer with Alexa Fluor 488 annexin V and propidium iodide (PI). After the incubation, flow cytometry was carried out to measure the fluorescent intensity of the stained cells by an imaging flow cytometer (Amnis Corporation, WA, USA).

#### Prolonging the plasma treatment time on medium to shorten the PAM treatment time on cells

To evaluate the possibility of decreasing the PAM treatment time for use in clinical applications, we increased the plasma exposure time in order to increase the RONS concentration in PAM. Exposure times of 2, 4, and 6 min were used, cells were treated with PAM for 30, 60, 90, and 120 min, and their viability was examined using an MTT assay.

#### Comparison of PAM therapy with hyperthermochemotherapy

Hyperthermochemotherapy was performed with water at 43 °C supplemented with 200 mg/m^3^ cisplatin. Cells were seeded on a 96-well culture plate 1 day before the treatment, and then treated with 43 °C cisplatin-added water for 2 h. The therapeutic effects were evaluated by measuring the viability using an MTT assay. The results for treatment with 43 °C cisplatin-added water were compared with those for PAM treatment.

#### FGF-1 and TGF-β1 concentration after PAM treatment

To investigate if PAM inhibits cancer cell growth by affecting FGF-1 and TGF-β1, CL1–5 cells were cocultured with CAF cells and treated with PAM. Then 2 × 10^5^ CL1–5 cells were cultured individually or cocultured with 1.5 × 10^5^ CAF cells in a 24-well plate with polycarbonate cell culture inserts (Nunc, Thermo Fisher Scientific, USA). The individually cultured and cocultured cells were then treated with PRMI medium and PAM for 2 h. After replacing the medium and allowing another day of culturing, the FGF-1 and TGF-β1 concentration in conditioned medium was measured using a human FGF-1 ELISA kit (Thermo Fisher Scientific) and TGF β1 human/mouse uncoated ELISA kit (Invitrogen, Thermo Fisher Scientific, USA), respectively, via colorimetric reactions.

#### Statistical analysis

We analyzed the differences between two groups using Student’s *t* test and the differences are considered to be statistically significant when *P* < 0.05.

## Results

### OES of free NTAPPJ

**Figure 2:**
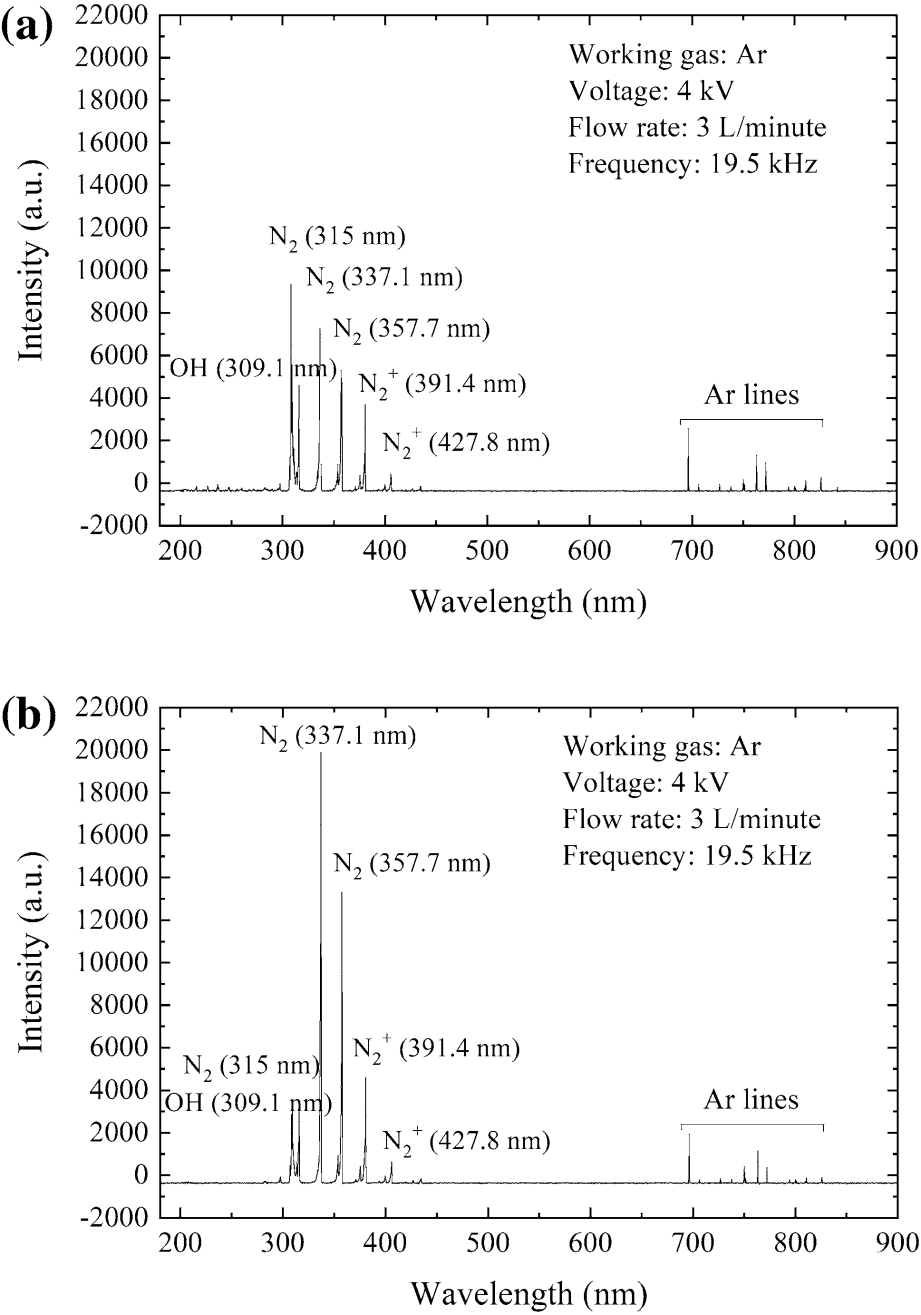
OES of free NTAPPJ in air (**a**) and 2 mm above the NTAPPJ–liquid interface (**b**).

The species in the postdischarge region of the free NTAPPJ and at the NTAPPJ–medium interface was analyzed based on the measured OES (Fig. 2). The spectrum revealed the emission intensities of RONS, including ^·^OH radicals, N_2_, and N_2_^+^, and of the activated Ar from working gas. The intensity of the ^·^OH-radical emission was significantly enhanced on the liquid surface, which might have been due to the dissociation of water molecules.

### Measurement of RONS in solutions

Numerous studies have demonstrated that plasma exposure is effective at inducing apoptosis in a broad range of cancer cells while exerting only minor effects on normal cells^43,44^. The main factors underlying these effects are considered to be RONS such as ^·^OH radicals and ONOO^–^^45^. It is particularly interesting that indirectly treating cancer cells with PAM reportedly has similar effects to direct plasma treatments^46,47^, which may broaden the therapeutic application of plasma for therapies in the body. We therefore measured the concentrations of RONS in PAM in an attempt to understand the effects of the RONS dosage.

#### ^·^OH radicals generated by NTAPPJ

**Figure 3:**
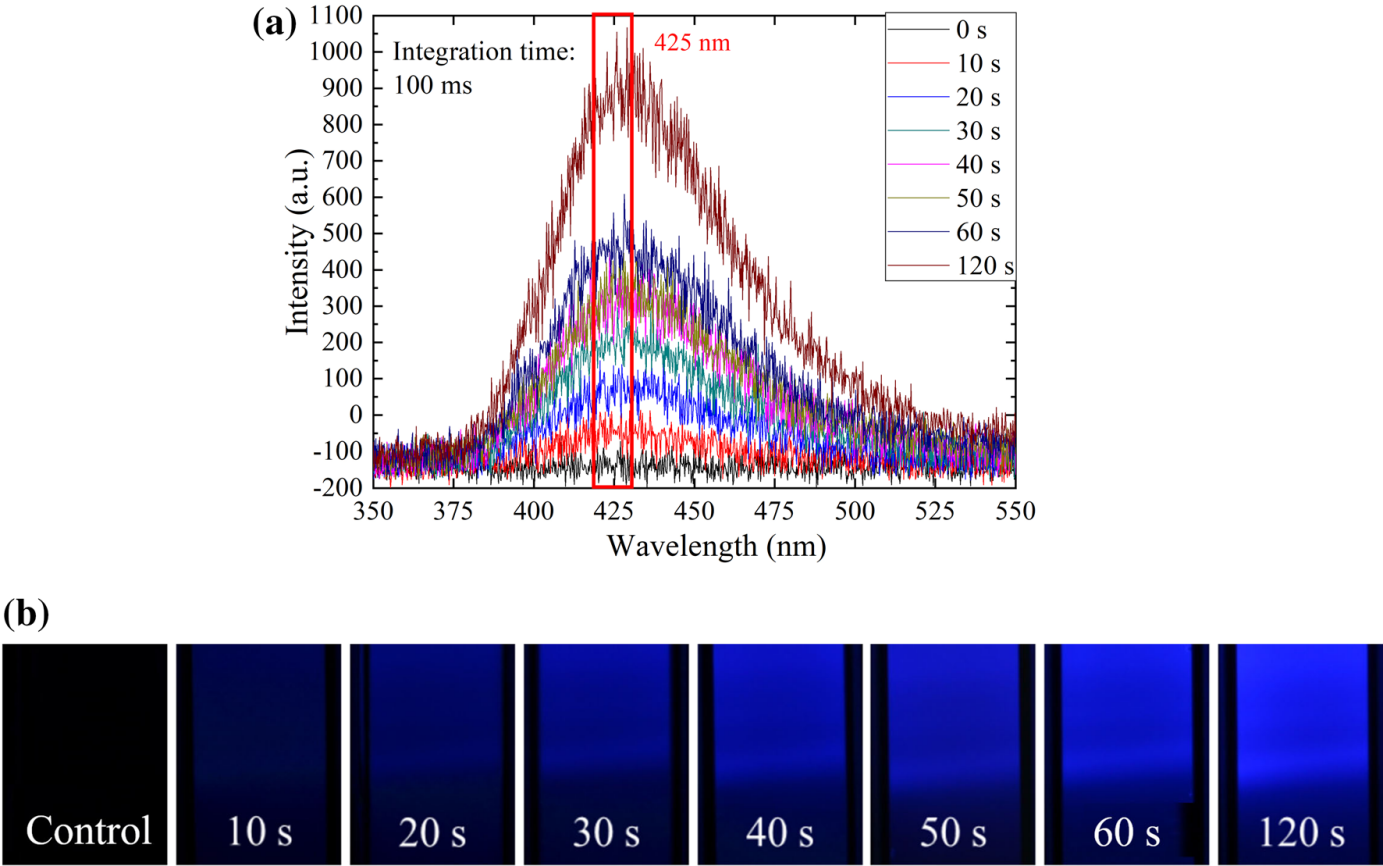
RPMI medium was exposed to NTAPPJ for various time periods. The fluorescent intensity of HTA was measured using an OES (**a**), with images taken using a CCD camera (**b**).

^·^OH radicals play a key role in the plasma effects on cancer cells and are related to other plasma species that can influence cancer cells, such as ONOO^–^ and O_2_^–^ radicals^45^. Hence, we measured the ^·^OH radicals generated in the NTAPPJ-treated solution, based on the reaction with TA. The fluorescent intensities and images of the reaction product, HTA, were recorded as an OES and using a CCD camera (Fig. 3). In creasing the exposure time to NTAPPJ increased the 425-nm intensity, which revealed the accumulation of HTA increased with exposure time.

#### H_2_O_2_ concentration in PAM and PAM-RPMI mixtures

**Figure 4:**
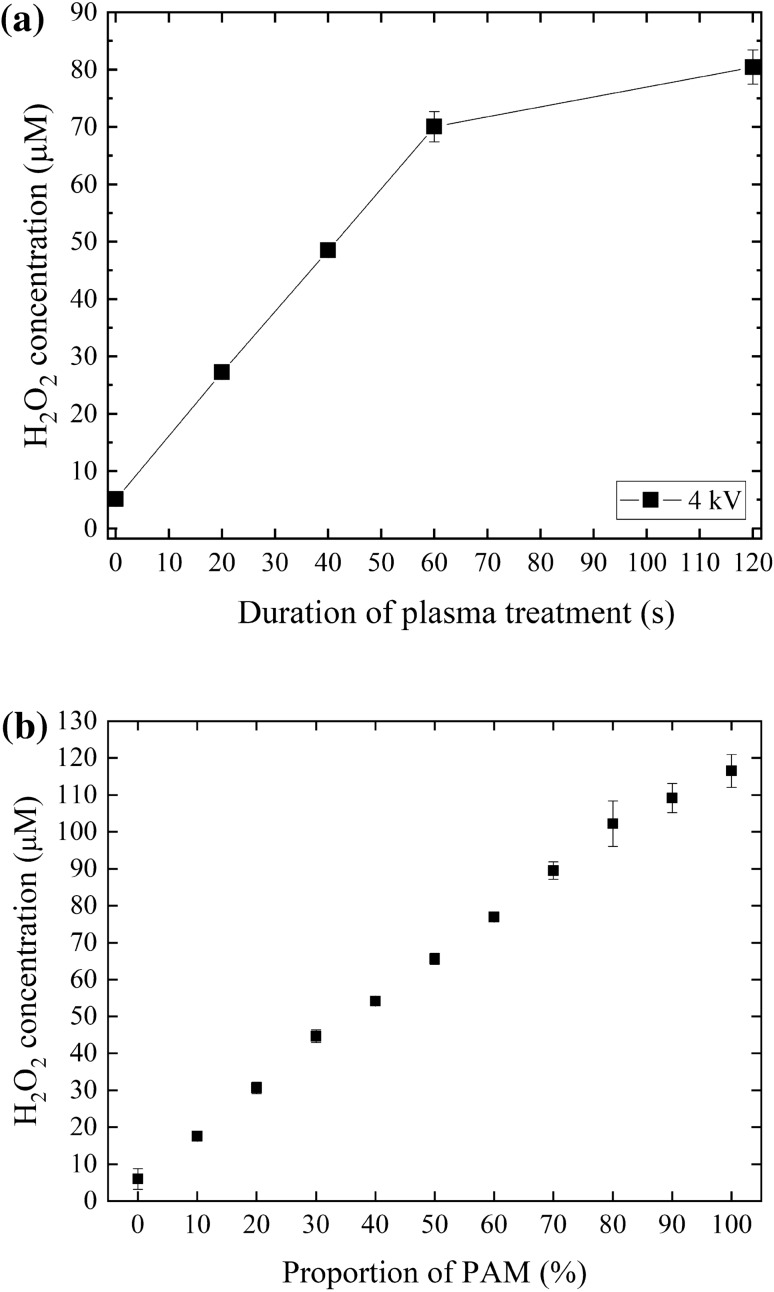
(**a**) H_2_O_2_ concentration in PAM prepared with different exposure times. (**b**) PAM produced using a 120-s exposure time was serially diluted with untreated RPMI medium, and the H_2_O_2_ concentrations were measured for PAM present at different proportions. Data are mean and SD values (n = 3).

RPMI medium without phenol red was exposed to NTAPPJ, and the generated H_2_O_2_ was measured using an Amplex Red assay kit (Fig. 4). The H_2_O_2_ concentration increased linearly as the exposure time increased from 0 to 60 s, with this increase subsequently slowing down to enter a plateau (Fig. 4a). To control the concentration of RONS in PAM for treatments, PAM prepared using an exposure time of 120 s was serially diluted with untreated RPMI medium to produce PAM-RPMI mixtures with different proportions of PAM. The H_2_O_2_ concentration increased linearly with the proportion of PAM (Fig. 4b), which suggests that the concentrations of other ROS in the PAM-RPMI mixture could also change linearly with the proportion of PAM.

#### Nitrite concentration in PAM-RPMI mixtures

**Figure 5:**
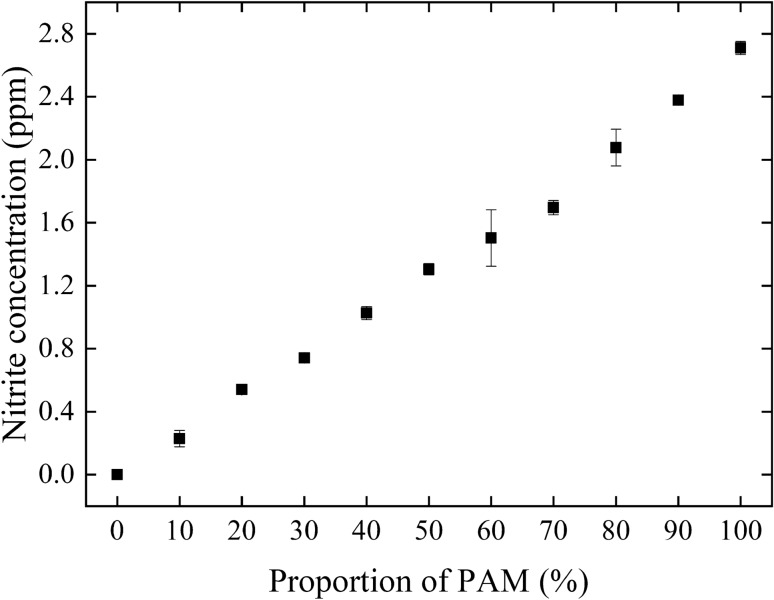
PAM prepared with a plasma exposure time of 120 s was serial diluted with untreated RPMI medium. The nitrite concentrations were measured for PAM present at different proportions. Data are mean and SD values (n = 3).

The nitrite concentration in the PAM-RPMI mixtures was measured using the Griess test, and was found to increase linearly with the proportion of PAM (Fig. 5). It suggests that the concentrations of some other RNS in the PAM-RPMI mixture may also change linearly with the proportion of PAM.

### Effects of PAM on cancer and benign cells

To clarify the possibility of applying PAM as a lung cancer therapy, we investigated the antitumor effects of PAM on lung cancer cells. The effects of PAM on benign cells were also analyzed and compared.

#### Effects of PAM on cell viability, morphology, and apoptosis

**Figure 6:**
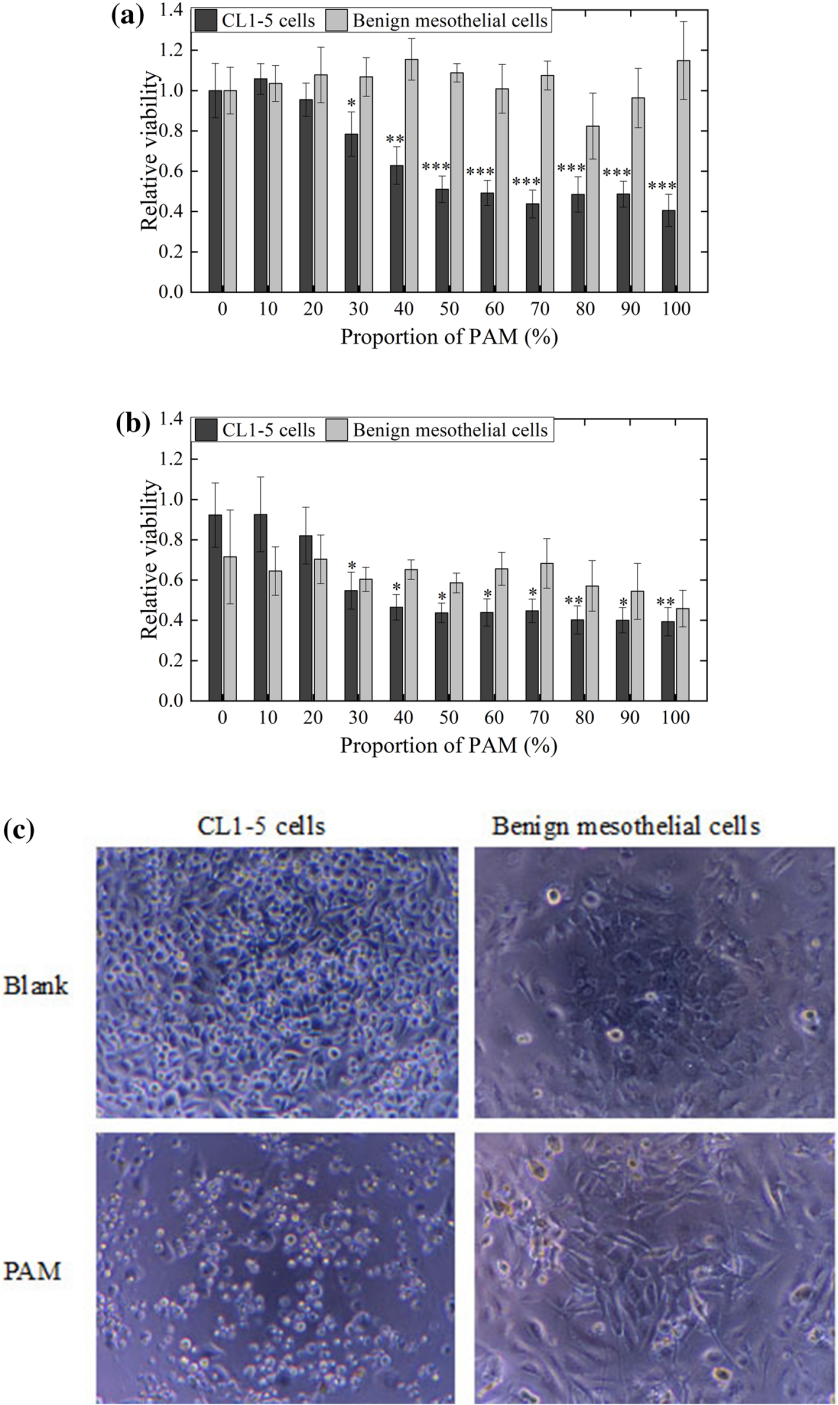

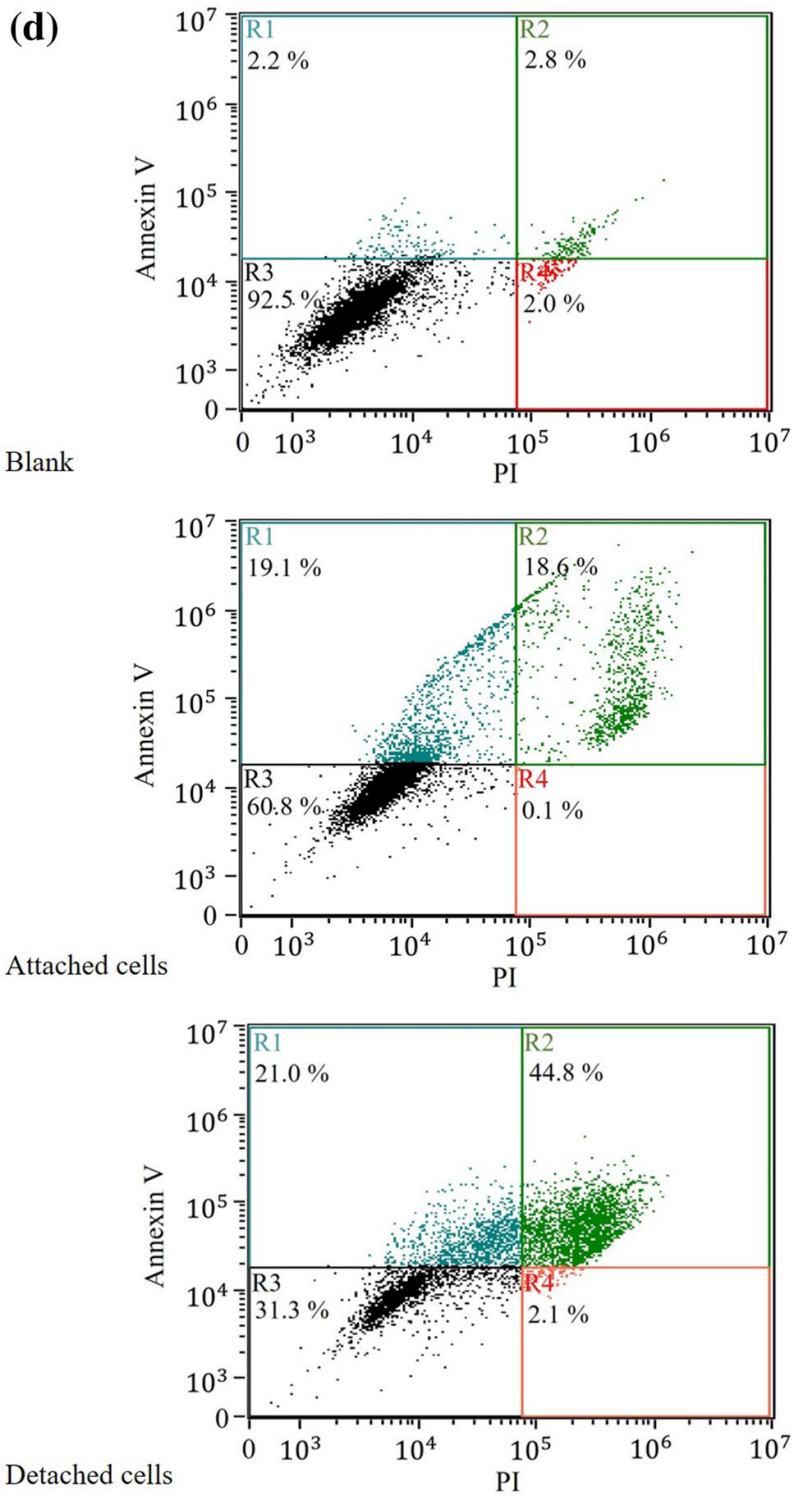
CL1–5 cells and primary cultured benign mesothelial cells were treated with PAM at 37 °C (**a**) or 43 °C (**b**) for 2 h. The relative viability is shown comparing to the treatment of 0% PAM (RPMI without plasma treatment) at 37 °C. The MTT assay was performed after further culturing for 1 day. Data are mean and SD values (n ≥ 3). * *P* < 0.05; ** *P* < 0.01; *** *P* < 0.001 (Student’s *t* test). (**c**) Photographs of CL1–5 and benign mesothelial cells before and 1 day after receiving treatment with PAM for 2 h. (**d**) The attached and detached CL1–5 cells after PAM treatment were harvested separately. After the dual staining of Annexin V-Alexia 488 and PI, the apoptosis of CL1–5 cells with (attached cells and detached cells) and without (blank) PAM treatments was analyzed by flow cytometry.

Lung cancer CL1–5 cells and primary cultured benign mesothelial cells were treated with PAM at 37 °C or 43 °C for 2 h. After replacing the PAM with fresh medium, cells were kept in the culture for a further 1 day. Cell viability was assessed using an MTT assay. At 37 °C, the viability of CL1–5 cells decreased when the proportion of PAM (dosage) increased (Fig. 6a). In contrast, the viability of benign mesothelial cells was affected by PAM under the same condition. When the proportion of PAM exceeded 50% (H_2_O_2_ ~ 60; nitrite ~ 1.2 ppm), there was a significant difference in the viabilities of the two types of cells. This indicates that PAM exerts selective lethal effects on CL1–5 and benign mesothelial cells. Comparatively, the viabilities of both CL1–5 and benign mesothelial cells were clearly decreased after the treatment at 43 °C (Fig. 6b), from which we speculate that the clinical thermotherapy had a side effect of damaging normal cells in the flow path of the 43 °C water.

The morphologies of CL1–5 and benign mesothelial cells was observed before and 1 day after treatments with PAM for 2 h (Fig. 6c). The confluency of CL1–5 cells decreased significantly after PAM treatment. A large proportion of the CL1–5 cells shrank and exhibited the characteristics of apoptosis. Benign mesothelial cells also showed some morphology changes, but their coverage of the culture area did not have change significantly. Combined with the results of the MTT assay, most of the benign mesothelial cells survived being treated with PAM.

The apoptotic CL1–5 cell death induced by PAM treatments was analyzed using the dual labeling of Annexin V and PI with flow cytometry 18 h after the treatments (Fig. 6d). The detached cells and attached cells after the treatments were harvested separately. The percentages of population R1 referred to as early apoptosis cells and R2 referred to as late apoptosis cells of PAM-treated cells significantly were higher in comparison with the cells without PAM treatments. Meanwhile, the percentages of population R4 referred to as non-apoptosis dead cells did not show obvious change. These results indicated the cancer cells underwent lethal stress which induced apoptosis during the treatment.

#### Effects of PAM on cell proliferation and mobility

**Figure 7:**
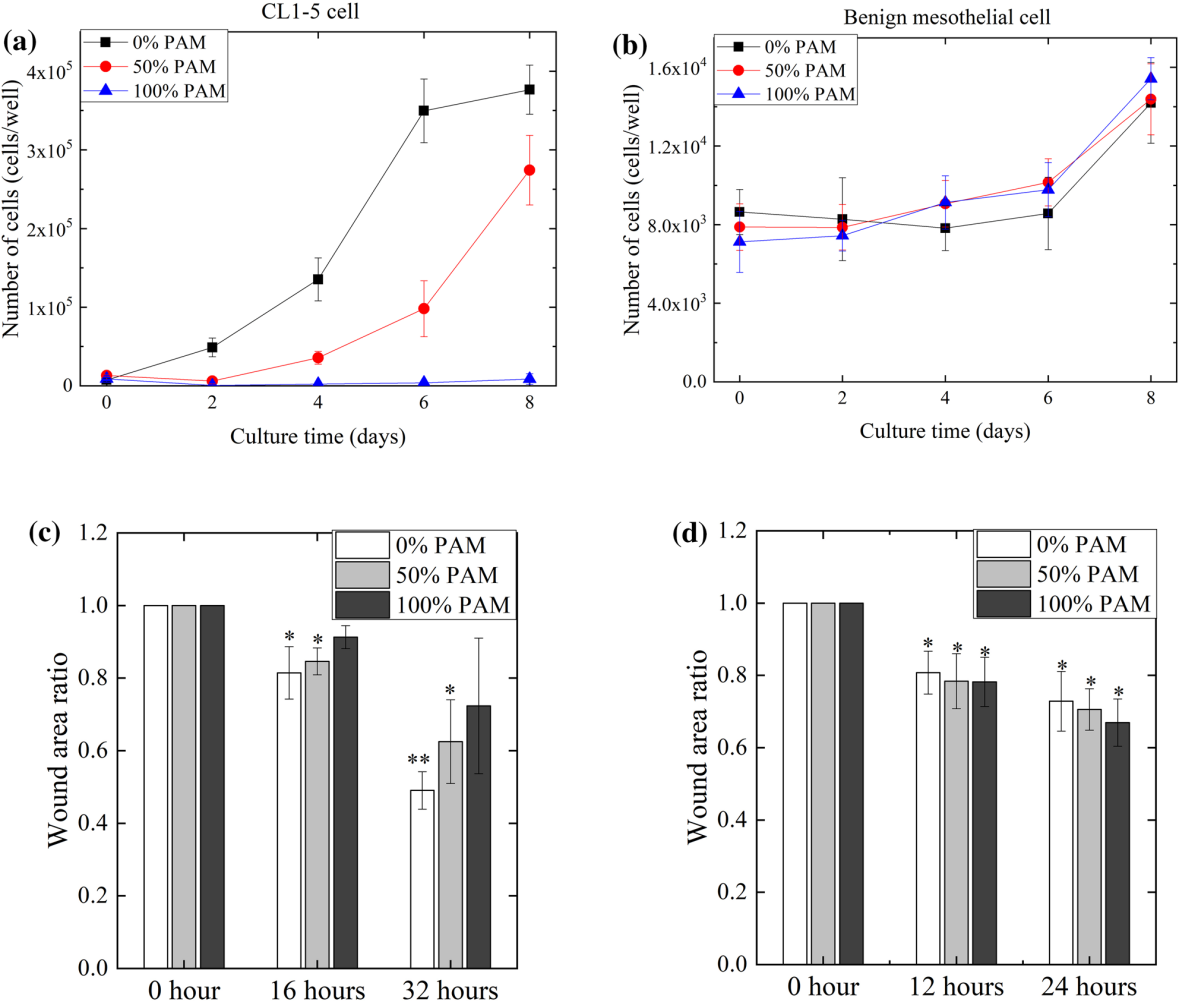
Proliferation of CL1–5 (**a**) and benign mesothelial (**b**) cells after treatment with PAM. The numbers of living cells were counted on days 0, 2, 4, 6, and 8. Data are mean and SD values (n = 3). Mobility of CL1–5 (**c**) and benign mesothelial (d) cells after the treatment with PAM was determined using a scratch assay. Data are mean and SD values (n = 3). * *P* < 0.05; ** *P* < 0.01 (Student’s *t* test).

Cells were observed and counted on every second day after the treatment with PAM for a total of 8 days. Living cells were counted using the trypan blue exclusion method. While the number of untreated CL1–5 cells increased about 20-fold from day 0 to 8, there was no increase in the number of cells treated with 100% PAM (Fig. 7a). Even treatment with 50% PAM was effective at reducing the number of CL1–5 cells. Nevertheless, the proliferation of benign mesothelial cells did not reveal clear differences between the untreated and treated cells (Fig. 7b).

The effects of PAM on cell mobility were determined using a scratch assay. ImageJ was used to quantify the wound area at 16 and 32 h after the treatment with PAM. CL1–5 migrated to cover about 50% of the wound area after 32 h when treated with 0% PAM, with this migration increasing to cover an average of 70% when treated with 100% PAM (Fig. 7c). That means PAM decreased the mobility of CL1–5 cells. This effect was not found in benign mesothelial cells until 24 h after PAM treatment (Fig. 7d).

#### Effects of PAM on the viability of various cell types

**Figure 8:**
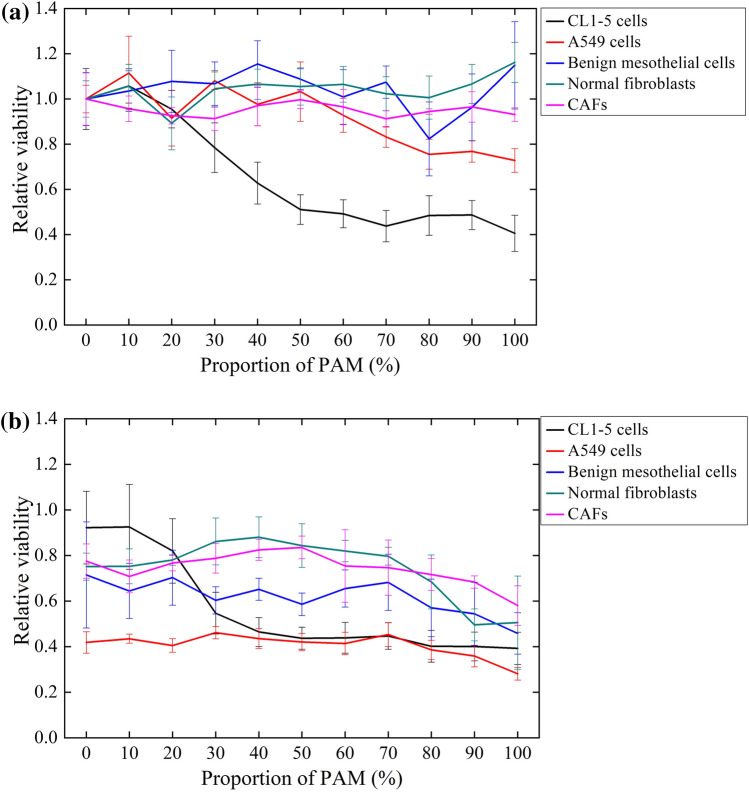
Various cell types were treated with PAM at 37 °C (**a**) and 43 °C (**b**) for 2 h. Relative viabilities are calculated comparing to the treatment of 0% PAM (RPMI without plasma treatment) at 37 °C. Cell viability was analyzed using an MTT assay at 1 day after the treatment. Data are mean and SD values (n = 3).

To further investigate the effects of PAM on cancer therapy (in addition to those on CL1–5 and benign mesothelial cells), several cancer and benign cells—including A549 cells, normal fibroblasts, and CAFs—were also treated with PAM at 37 °C and 43 °C, and their cell viabilities were analyzed (Fig. 8). The results showed that PAM was selectively toxic to CL1–5 and A549 cancer cells at 37 °C (Fig. 8a), with CL1–5 cells being more sensitive than A549 cells at the same dose. At 43 °C, all of the treated cells were obviously damaged. (Fig. 8b) A549 cells were more sensitive to thermal treatment than CL1–5 cells were for low-dose PAM.

### Comparison of PAM treatment with hyperthermochemotherapy

**Figure 9:**
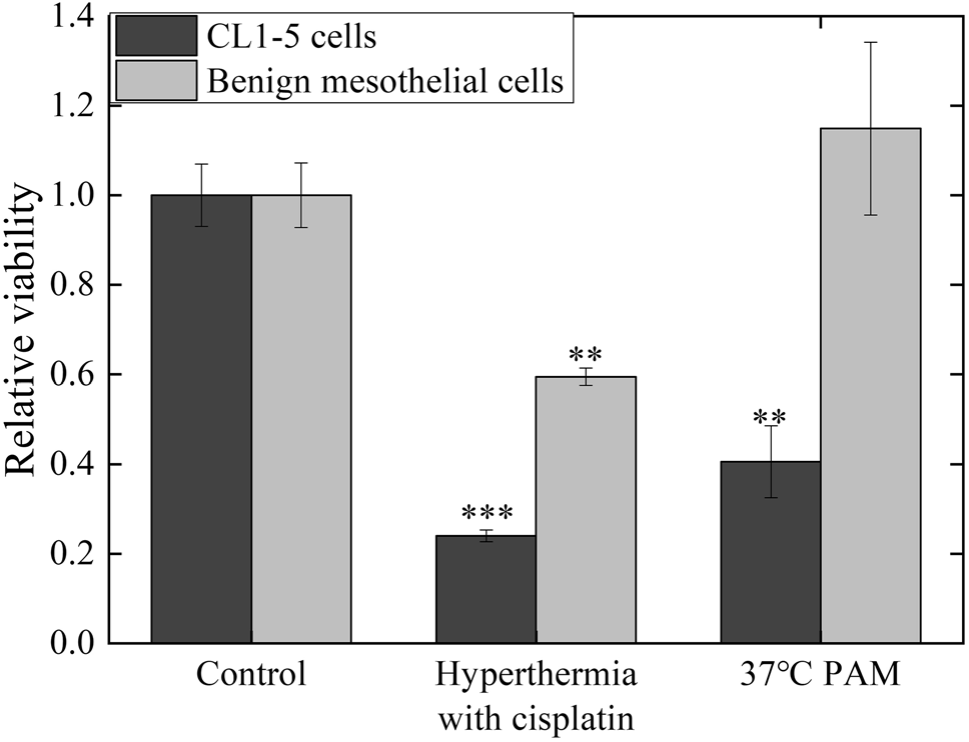
CL1–5 and benign mesothelial cells were treated with 100% PAM at 37 °C or saline with 200 mg/m^2^ cisplatin at 43 °C for 2 h. An MTT assay was performed to determine the cell viability. Data are mean and SD values (n = 3). * *P* < 0.05; ** *P* < 0.01; ***P < 0.001 (Student’s *t* test).

The treatment with PAM was demonstrated to selectively exert inhibition effects on cancer cells. To further refine the possibility of applying PAM in clinical cancer therapy, we compared the efficacy of PAM treatment with that of clinically applied hyperthermochemotherapy. The imitative hyperthermochemotherapy was carried out by treating CL1–5 and benign mesothelial cells with 200 mg/m^2^ cisplatin in saline at 43 °C for 2 h^8,9^. The cell viability was determined using an MTT assay (Fig. 9). Treating hyperthermia with cisplatin was efficient at killing CL1–5 cells, but also seriously damaged benign mesothelial cells, which might be indicative of the side effects caused by hyperthermochemotherapy. In contrast, the viability of benign mesothelial cells was significantly greater for PAM treatment than for hyperthermochemotherapy, which implies that PAM treatment has fewer side effects than hyperthermochemotherapy.

### Reducing the PAM treatment time by prolonging PAM exposure time to plasma

**Figure 10:**
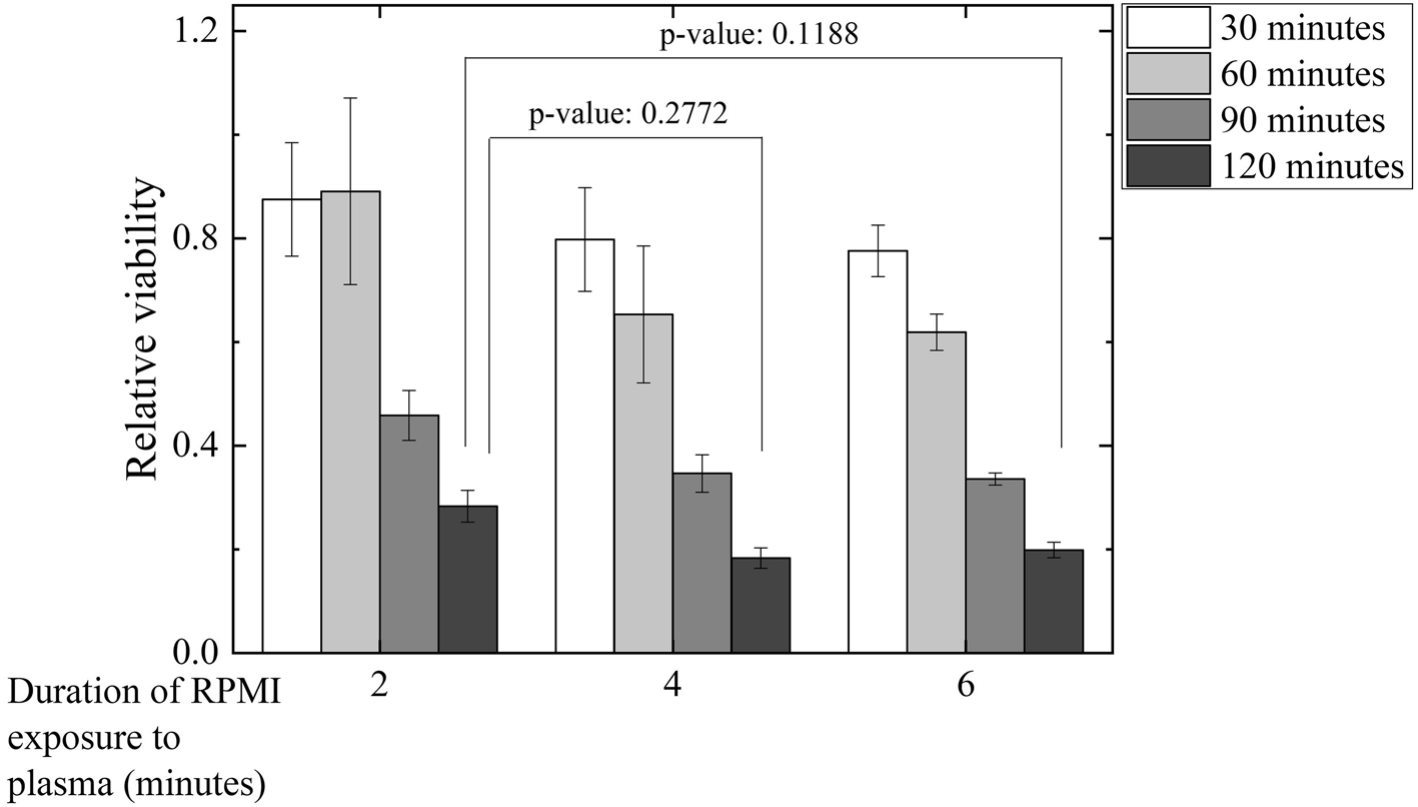
CL1–5 cells were treated with PAM for 30, 60, 90, and 120 min at 37 °C. An MTT assay was performed 1 day after the treatments. RPMI medium was exposed to NTAPPJ for 2, 4, and 6 min to prepare PAM. Data are mean and SD values (n = 3). (Student’s *t* test).

A clinical course of hyperthermochemotherapy usually takes 2 h, which can be very stressful for patients because of the high temperature and long treatment time. Therefore, we considered the possibility of reducing the treatment time by applying PAM at a higher dose. We prepared PAM at higher doses by increasing the exposure time of NTAPPJ to RPMI medium from 2 min to 4 and 6 min. CL1–5 cells were treated with PAM for 30, 60, 90, and 120 min at 37 °C. An MTT assay was performed 1 day after the treatments (Fig. 10). Increasing the exposure time to produce PAM with higher dose increased the lethal effects. Meanwhile, 4- and 6-min PAM treatments for 90 min produced similar lethal effects to 2-min PAM treatment for 120 min with p-value of 0.2772 and 0.1188, respectively. This indicates that the treatment time could be decreased by increasing the PAM exposure time to plasma.

### Effects of PAM on the tumor microenvironment

**Figure 11:**
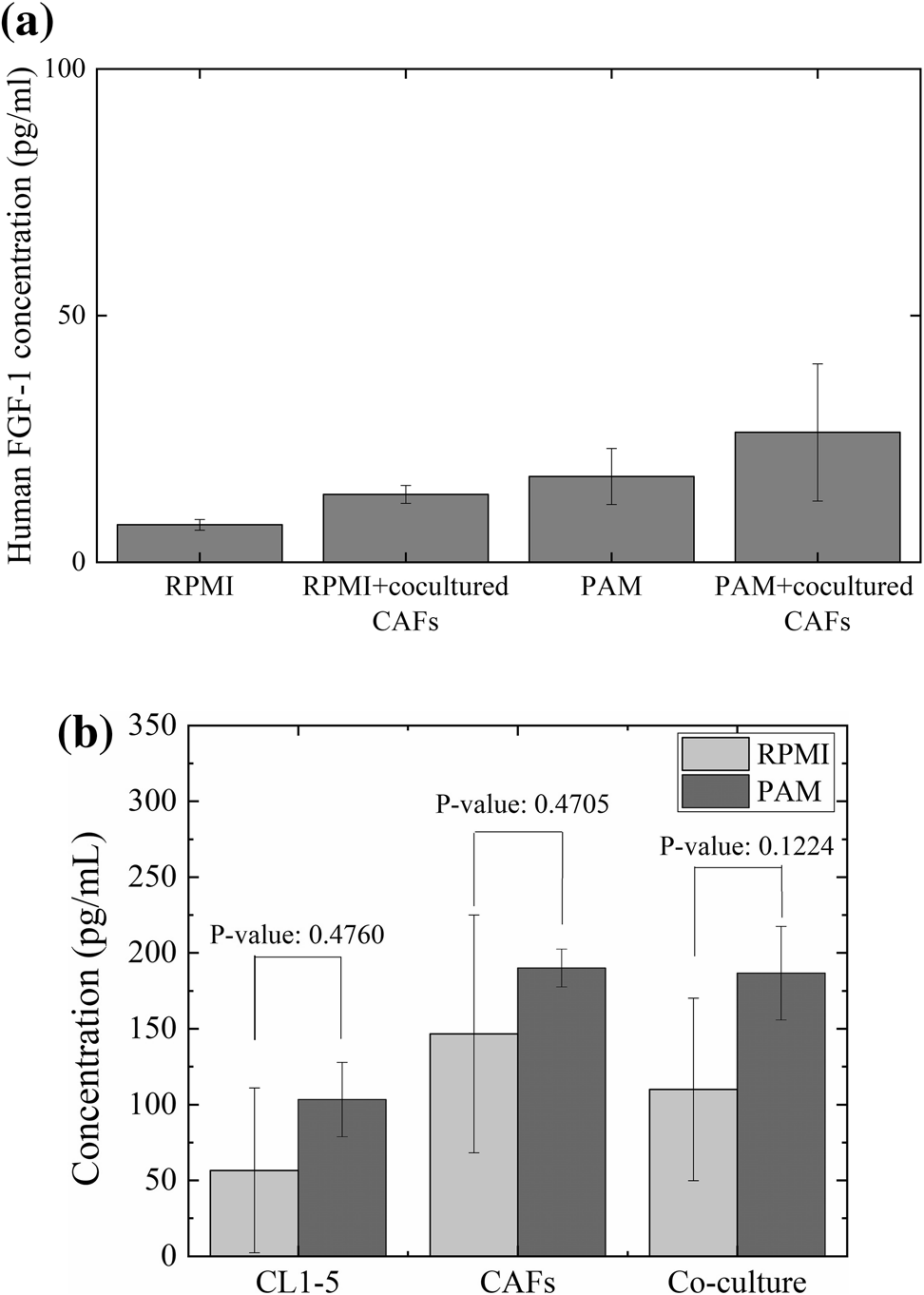
CL1–5 cells were cultured individually or cocultured with CAFs and treated with normal RPMI medium or PAM. The FGF-1 (**a**) and TGF-β1 (**b**) concentration in conditioned medium was analyzed using an FGF-1 ELISA kit. Data are mean and SD values (n = 3). (Student’s *t* test).

This study also measured the FGF-1 and TGF-β1 concentration after PAM treatment, in an attempt to determine why PAM can selectively damage CL1–5 cancer cells. Fibroblast growth factors such as FGF-1 can be secreted by CAFs to promote tumorigenesis of many different kinds of cancers^48^. To investigate whether the PAM treatment changes only cancer cells themselves or also the tumor environment, CL1–5 cells were cultured individually or cocultured with CAFs, and then treated with the RPMI medium or PAM. The concentration of human FGF-1 and TGF-β1 in the conditioned medium was detected and quantified (Fig. 11). The results showed that the FGF-1 concentrations were higher in both cocultured systems, which was consistent with the previous finding that CAFs secrete FGF-1^48^. We also found the PAM treatment slightly increased the CL1–5 secretion of FGF-1. Nevertheless, the FGF-1 concentration was always lower than 50 pg/mL in our study, which was lower than the effective FGF-1 concentration (about 400 pg/mL)^49^. Hence, PAM can selectively inhibit CL1–5, but not by mediating FGF-1 secretion.

On the other hand, the concentration of TGF-β1 in conditioned media from CAFs was a little higher than it from CL1–5 (*P* = 0.25) but similar to it from cocultured cells (*P* = 0.63) after RPMI treatments. This also indicated the TGF-β1 amount in cocultured amount majorly attributed to the secretion of CAFs. The treatments of PAM slightly increased the concentration of TGF-β1 weather in monocultured or cocultured cells in comparison to RPMI treatments.

## Discussion

The NTAPPJ used in this study generated RONS in free jet and in an RPMI medium. The intensity of ^·^OH-radical emissions increased at the liquid–plasma interface, which was inferred to be due to the dissociation of water molecules. ^·^OH radicals enter the liquid and generate H_2_O_2_ or ONOO^–^, and ONOO^–^ is reported to be the important factor for cancer cell apoptosis^45^. From our results, the ^·^OH radicals kept generating and reacted with TA to result in the accumulation of HTA when the exposure time of the liquid to NTAPPJ was increased from 0 to 120 s. It indicates effective PAM can be generated.

Based on the limitation of current technology for chemical analysis, there might be effective RONS in PAM for cancer therapy that are currently unknown. However, PAM has revealed its advantages for medical applications. To facilitate plasma clinical applications, measuring the concentrations of all of the RONS might require an objective and easy way to identify the dosage of PAM. Here we used H_2_O_2_ and nitrite concentration as the references of RONS concentration. Our results indicated that the concentrations of H_2_O_2_ and nitrite varied linearly with the proportion of PAM in the PAM-RPMI mixtures, which might further conjectures that the concentrations of H_2_O_2_ and nitrite can be used as easy and fast indexes of the long-life RONS dosage of PAM. Also, H_2_O_2_ and nitrite are known as two effective factors for cancer cell inhibition. In this study, we used proportionally diluted PAM-RPMI mixture instead of different NTAPPJ treatment time, so that the RONS concentration applied to the cells can be carefully controlled.

Hyperthermia with cisplatin exerts strong MPE therapeutic effects, but it can also cause side effects. When applied at appropriate dosages, PAM facilitated the lethality to cancer cells while there were only minor effects on benign cells in our study. These observations represent strong evidence of the possibility of applying PAM in lung cancer therapy. Moreover, the mobility of cancer cells was inhibited by PAM, and so treatment with PAM is a promising method for repressing cancer cell metastasis. From our observations of cell morphology, we conjecture that the lethality caused by PAM occurred largely via apoptosis. Although apoptosis is not always noninflammatory, in comparison with the lethality via cell necrosis that can trigger inflammation, the application of PAM in cancer therapy could be a better option. Since the selective lethality of PAM is effective at 37 °C and that the lethal effects of PAM are even stronger than thermal treatment with normal RPMI medium, it might be possible to treat patients with plural effusion using PAM at 37 °C, which would avoid the endurance required for high-temperature thermotherapy.

Many studies have found that CAFs are involved in the crosstalk in tumor microenvironments. CAFs can secrete many kinds of protein factors (e.g., VEGF, FGF-1, TGF-β, and IL-6) to promote the proliferation of tumor cells, the angiogenesis around tumors, and the invasion and metastasis of tumors^50^. Furthermore, the ROS level and and TGF-β signaling may have interaction in lung cancer^51^. To take a further step to clarify the effects of PAM in tumor microenvironments, we also investigated whether PAM affects the secretion of FGF-1 and TGF-β1. In a result, PAM induced the slight secretion of both FGF-1 and TGF-β1. The amount detected in our study was 10 times lower than the effective FGF-1 concentration reported previously (about 400 pg/mL)^49^. Therefore, we assume the selective cytotoxicity of PAM to cancer cells is not through its effect on FGF-1 secretion. On the other hand, the signaling of TGF-β is considered to promote cancer metastasis in advanced cancers and in tumor environment. However, one assumption of the selective effect caused by plasma treatment is the differently basal cellular ROS level between cancer and normal cells^52^. When the inherent ROS in cancer cells is closer to the lethal threshold, cancer cells are more susceptible to oxidative stress. TGF-β can also induce redox imbalance by raising ROS/RNS production^53^. It is still difficult to conclude if the slightly increased TGF-β1 after PAM treatment involves in PAM-induced therapeutic selectivity or resistance.

Our results show that using PAM with a higher concentration, as prepared by increasing the exposure time to NTAPPJ, has the possibility of decreasing the treatment time or improving the therapeutic effects. However, different cancer cells exhibit different responses or sensitivities to PAM. Further studies are therefore required to obtain a deeper understanding of the underlying mechanisms and to optimize the curative programs. Along with the development of precision medicine, the specific tissues, genetic and cellular backgrounds, and other possible factors will need to be considered in decision-making about treatments. More appropriate and effective treatments should be applied to patients to improve the therapeutic quality, and PAM has clinical potential as an adjuvant or therapeutic formula.

## Conclusion

This study has investigated the efficacy of NTAPPJ in lung cancer therapy. The concentrations of RONS related to cancer therapy such as NO_2_^–^, in addition to H_2_O_2_ in PAM and ^·^OH radicals in solution were measured. The selective effects of PAM on cell viability, proliferation, morphology, and migration were studied and compared between lung cancer cells and benign cells. A coculture system was utilized to analyze the effects of PAM on the secretion of FGF-1 by CAFs, with the aim of understanding the change in the tumor environment when PAM is applied as a therapeutic agent.

Compared to hyperthermochemotherapy, treatment with PAM has the potential to effectively decrease the high-temperature burden experienced by patients suffering from plural effusion caused by lung cancer. We expect that a plasma-based malignant-pleural-effusion therapy will be established to solve the problems of cisplatin and PDT therapy.

## References

[1] Jemal A (2011). Global cancer statistics. CA Cancer J. Clin..

[2] Institute, N. C. *Cancer stat facts: leukemia*. https://seer.cancer.gov/statfacts/html/leuks.html (2020).

[3] Yasufuku K, Fujisawa T (2007). Staging and diagnosis of non-small cell lung cancer: invasive modalities. Respirology.

[4] Robert M (2010). Murray and Nadel's Textbook of Respiratory Medicine.

[5] Wang JS, Tseng CH (1995). Changes in pulmonary mechanics and gas exchange after thoracentesis on patients with inversion of a hemidiaphragm secondary to large pleural effusion. Chest.

[6] Porcel JM (2015). Clinical features and survival of lung cancer patients with pleural effusions. Respirology.

[7] Mattson K (1992). Multimodality treatment programs for malignant pleural mesothelioma using high-dose hemithorax irradiation. Int. J. Radiat..

[8] Matsuzaki Y (1984). Experimental studies of hyperthermia against MH134 tumor on mice. Antitumor effects under various conditions of heating. Nihon Geka Gakkai zasshi..

[9] Matsuzaki Y (1991). Thermotolerance in regional hyperthermiain vivo. Jpn. J. Surg..

[10] Matsuzaki Y (1995). Intrapleural perfusion hyperthermo-chemotherapy for malignant pleural dissemination and effusion. Ann. Cardiothorac. Surg..

[11] Shigemura N (2004). Pleural perfusion thermo-chemotherapy under VATS: a new less invasive modality for advanced lung cancer with pleural spread. Ann. Cardiothorac. Surg..

[12] Baas P (1997). Photodynamic therapy as adjuvant therapy in surgically treated pleural malignancies. Br. J. Cancer.

[13] Friedberg JS (2004). Phase II trial of pleural photodynamic therapy and surgery for patients with non–small-cell lung cancer with pleural spread. J. Clin. Oncol..

[14] Kalghatgi S (2011). Effects of non-thermal plasma on mammalian cells. PLoS ONE.

[15] Panngom K (2013). Preferential killing of human lung cancer cell lines with mitochondrial dysfunction by nonthermal dielectric barrier discharge plasma. Cell Death Dis..

[16] Yadav DK (2020). Cold atmospheric plasma generated reactive species aided inhibitory effects on human melanoma cells: an in vitro and in silico study. Sci. Rep..

[17] Keidar M (2018). A prospectus on innovations in the plasma treatment of cancer. Phys. Plasmas.

[18] Keidar M (2011). Cold plasma selectivity and the possibility of a paradigm shift in cancer therapy. Br. J. Cancer.

[19] Partecke LI (2012). Tissue tolerable plasma (TTP) induces apoptosis in pancreatic cancer cells in vitro and in vivo. BMC Cancer.

[20] Tanaka H (2015). Cancer therapy using non-thermal atmospheric pressure plasma with ultra-high electron density. Phys. Plasmas..

[21] Utsumi F (2013). Effect of indirect nonequilibrium atmospheric pressure plasma on anti-proliferative activity against chronic chemo-resistant ovarian cancer cells in vitro and in vivo. PLoS ONE.

[22] Tanaka H (2011). Plasma-activated medium selectively kills glioblastoma brain tumor cells by down-regulating a survival signaling molecule, AKT kinase. Plasma Med..

[23] Kaushik NK (2018). Biological and medical applications of plasma-activated media, water and solutions. Biol. Chem..

[24] Adachi T (2016). Iron stimulates plasma-activated medium-induced A549 cell injury. Sci. Rep..

[25] Adachi T (2015). Plasma-activated medium induces A549 cell injury via a spiral apoptotic cascade involving the mitochondrial–nuclear network. Free Radic. Biol. Med..

[26] Hara H, Taniguchi M, Kobayashi M, Kamiya T, Adachi T (2015). Plasma-activated medium-induced intracellular zinc liberation causes death of SH-SY5Y cells. Arch. Biochem. Biophys..

[27] Huang J (2011). Deactivation of A549 cancer cells in vitro by a dielectric barrier discharge plasma needle. J. Appl. Phys..

[28] Huang J (2011). Dielectric barrier discharge plasma in Ar/O_2_ promoting apoptosis behavior in A549 cancer cells. Appl. Phys. Lett..

[29] Xu D (2015). In situ OH generation from O_2_^−^ and H_2_O_2_ plays a critical role in plasma-induced cell death. PLoS ONE.

[30] Brüne B (2003). Nitric oxide: NO apoptosis or turning it ON?. Cell Death Differ..

[31] Chung HT, Pae HO, Choi BM, Billiar TR, Kim YM (2001). Nitric oxide as a bioregulator of apoptosis. Biochem. Biophys. Res. Commun..

[32] Yan X (2012). Plasma-induced death of HepG2 cancer cells: intracellular effects of reactive species. Plasma Process Polym..

[33] Kim SJ, Chung T (2015). Plasma effects on the generation of reactive oxygen and nitrogen species in cancer cells in-vitro exposed by atmospheric pressure pulsed plasma jets. Appl. Phys. Lett..

[34] Bauer G (2014). Targeting extracellular ROS signaling of tumor cells. Anticancer Res..

[35] Virág L, Szabo E, Gergely P, Szabo C (2003). Peroxynitrite-induced cytotoxicity: mechanism and opportunities for intervention. Toxicol. Lett..

[36] Adhikari M (2019). Cold atmospheric plasma and silymarin nanoemulsion synergistically inhibits human melanoma tumorigenesis via targeting HGF/c-MET downstream pathway. Cell Commun. Signal..

[37] Adhikari M, Adhikari B, Ghimire B, Baboota S, Choi EH (2020). Cold atmospheric plasma and silymarin nanoemulsion activate autophagy in human melanoma cells. Int. J. Mol. Sci..

[38] Keidar M, Yan D, Beilis II, Trink B, Sherman JH (2018). Plasmas for treating cancer: opportunities for adaptive and self-adaptive approaches. Trends Biotechnol..

[39] Fridman G (2008). Applied plasma medicine. Plasma Process. Polym..

[40] Laroussi M (2009). Low-temperature plasmas for medicine?. IEEE Trans. Plasma Sci..

[41] Chen W-J (2014). Cancer-associated fibroblasts regulate the plasticity of lung cancer stemness via paracrine signalling. Nat. Commun..

[42] Navab R (2011). Prognostic gene-expression signature of carcinoma-associated fibroblasts in non-small cell lung cancer. Proc. Natl. Acad. Sci..

[43] Kurake N (2016). Cell survival of glioblastoma grown in medium containing hydrogen peroxide and/or nitrite, or in plasma-activated medium. Arch. Biochem. Biophys..

[44] Wang M (2013). Cold atmospheric plasma for selectively ablating metastatic breast cancer cells. PLoS ONE.

[45] Graves DB (2012). The emerging role of reactive oxygen and nitrogen species in redox biology and some implications for plasma applications to medicine and biology. J. Phys. D.

[46] Chen CY, Cheng YC, Cheng YJ (2018). Synergistic effects of plasma-activated medium and chemotherapeutic drugs in cancer treatment. J. Phys. D Appl. Phys..

[47] Iseki S (2012). Selective killing of ovarian cancer cells through induction of apoptosis by nonequilibrium atmospheric pressure plasma. Appl. Phys. Lett..

[48] Sun Y (2017). Cancer-associated fibroblasts secrete FGF-1 to promote ovarian proliferation, migration, and invasion through the activation of FGF-1/FGFR4 signaling. Tumor Biology..

[49] Bai YP (2015). FGF-1/-3/FGFR 4 signaling in cancer-associated fibroblasts promotes tumor progression in colon cancer through Erk and MMP-7. Cancer Sci..

[50] Erdogan B, Webb DJ (2017). Cancer-associated fibroblasts modulate growth factor signaling and extracellular matrix remodeling to regulate tumor metastasis. Biochem. Soc. Trans..

[51] Cruz-Bermúdez A (2019). Cancer-associated fibroblasts modify lung cancer metabolism involving ROS and TGF-β signaling. Free Radic. Biol. Med..

[52] Trachootham D, Alexandre J, Huang P (2009). Targeting cancer cells by ROS-mediated mechanisms: a radical therapeutic approach?. Nat. Rev. Drug Discov..

[53] Liu R-M, Desai LP (2015). Reciprocal regulation of TGF-β and reactive oxygen species: a perverse cycle for fibrosis. Redox Biol..

